# Six year Trend in Cataract Surgical Techniques in Iran

**DOI:** 10.4103/0974-9233.80704

**Published:** 2011

**Authors:** Hassan Hashemi, Fatemeh Alipour, Shiva Mehravaran, Farhad Rezvan, Farshid Alaeddini, Akbar Fotouhi

**Affiliations:** 1 Department of Clinical Research, Noor Ophthalmology Research Center, Noor Eye Hospital, Tehran; 2 Farabi Eye Hospital, Tehran University of Medical Sciences, Tehran, Iran; 3 Health Researchers Research and Development Institute, Tehran, Iran; 4 Department of Epidemiology and Biostatistics, School of Public Health, Tehran University of Medical Sciences, Tehran, Iran

**Keywords:** Cataract Surgery, Extracapsular Cataract Extraction, Foldable Intraocular Lens, Intracapsular Cataract Extraction, Lensectomy Phacoemulsification

## Abstract

**Purpose:**

To determine the cataract surgery techniques performed in Iran from 2000 to 2005.

**Materials and Methods:**

This study was part of the Iranian Cataract Surgery Survey (ICSS) which was a retrospective cross-sectional study. All major ocular surgery units and 10% of randomly selected minor units throughout Iran were included. Excluding the 2 week Iranian New Year holiday, 1 week per season between 2000 and 2005 (a total of 24 weeks) was selected for each center, and data on all cataract surgeries performed during these weeks were collected by reviewing patient records. The ANOVA repeated measure test was performed to determine longitudinal changes with a P<0.05 denoting statistical significance.

**Results:**

Phacoemulsification with intraocular lens (IOL) implantation has become the surgical method of choice in Iran, increasing from less than 7% in 2000 to 57% in 2005 (P<0.0001). Extracapsular cataract extraction showed a reverse trend compared to phacoemulsification, decreasing from greater than 91% in 2000 to 41% in 2005 (P<0.0001). Intracapsular cataract extraction and lensectomy were rarely performed without significant changes over time (P>0.05).

**Conclusion:**

Phacoemulsification with IOL implantation has become the preferred cataract surgery method in Iran during recent years.

## INTRODUCTION

The history of cataract interventions dates back to more than 4000 years.^1^–^4^ Extracapsular cataract extraction. ECCE did not gain popularity until the mid-1950s when two significant advances occurred: microscopes were introduced to the operating room and Sir Harold Ridely replaced a cataractous lens with the first implanted intraocular lens (IOL) using the ECCE approach.^4^–^6^ The first phacoemulsification was done by Kelman in 1967, but it required the surgeon to learn special techniques and also expensive equipment, and therefore, ECCE continued to be the preferred method.^7^ Nonetheless, the obvious advantages of phacoemulsification, along with advanced instruments and better IOL designs, made it the method of choice among cataract surgeons in the 1980s, and is now commonly performed in developing countries as well.^8^,^9^

Cataract is responsible for 48% of global blindness and is among the most important causes of avoidable blindness that the “Vision 2020: The Right to Sight” initiative by the World Health Organization (WHO) is aiming to eliminate.^10^ The objective of this initiative warrants several strategies including not only promoting affordable and accessible cataract surgical services, but also improving the surgical quality to ensure satisfactory visual outcomes and quality of life for patients.^10^

To achieve the goals of Vision 2020, we designed a retrospective survey on the cataract surgical services performed throughout Iran, the Iranian Cataract Surgery Survey (ICSS). One of the main objectives of the ICSS was to determine the cataract surgical rate (CSR) the results of which have been published elsewhere.^11^ Here we report the methods of cataract surgery commonly performed in Iran yearly between 2000 to 2005. To the best of our knowledge, there are no other published reports on this subject.

## MATERIALS AND METHODS

In Iran, patients with cataracts resulting in significant reduction in visual acuity (usually less than 20/60) or other cataract-related symptoms such as significant glare or anisometropia are considered for surgery. The ICSS is a retrospective, descriptive, cross-sectional survey to report the annual number and type of cataract surgeries performed on the Iranian population of cataractous patients at cataract surgery units in Iran from 2000 to 2005. A detailed protocol and methodology of the study has been published elsewhere.^12^ A brief summary of the project is presented here.

Initially, all cataract surgery units (including university, government, military, private, etc) were identified throughout the country based on the report from the Ministry of Health. Cataract surgery units that performed more than 1000 cataract surgeries during the second half of 2004 were classified as “major” and others were grouped as “minor” cataract surgery units. All major units and 10% of randomly selected minor units were enrolled in the study. One week per season was randomly selected for each unit, excluding the first 2 weeks of spring which coincide with the Persian New Year holidays. For each unit, data concerning all cataract surgeries performed during a total of 24 weeks between 2000 and 2005 were recorded. The number of cataract surgeries performed in the representative 4 weeks was multiplied by 50/4 to calculate the total annual number of cataract surgeries for each center. The population information required for this study was gathered from the Iranian Statistics Center.

Data were extracted directly from patients’ surgical records archived at each center and recorded in standardized data forms by seven general practitioners who were trained specifically for data extraction via chart review for this study. All investigators worked closely with an ophthalmologist who was also a primary investigator in order to address any question and concerns regarding chart review. The first form included general information regarding the cataract surgery unit which was filled out by consulting the head of the ophthalmology department and the head of the operating rooms. The second form was completed by chart review of the patient records to collect data on examinations, type of surgery and details of the implanted IOL. The sampling design effect was taken into consideration in the calculation of 95% confidence intervals (CI) and the results were accordingly adjusted. *P* values less than 0.05 were considered statistically significant.

## RESULTS

A total of 13,409 records of cataract surgeries performed between 2000 and 2005 were reviewed from 28 centers. Nine centers were classified as major centers, and the rest were minor centers. Table 1 documents the number of cases reviewed at these centers and the estimated number of surgeries performed each year at minor centers and all major centers.

**Table 1:** Number of records reviewed at randomly selected minor cataract surgery units and all major cataract surgery units throughout the nation, with their yearly total from 2000 to 2005 and the estimated annual number of cataract surgeries performed at both types of units

Year	Reviewed records of CS			Estimated number of CS		
	Minor sites	Major sites	Total	Minor sites	Major sites	Total
2000	133	1335	1468	16625	16688	33313
2001	235	1428	1663	29375	17850	47225
2002	294	1763	2057	36750	22038	58788
2003	335	2186	2521	41875	27325	69200
2004	375	2472	2847	46875	30900	77775
2005	504	2349	2853	63000	29363	92363

CS denotes cataract surgery

**Figure 1:**
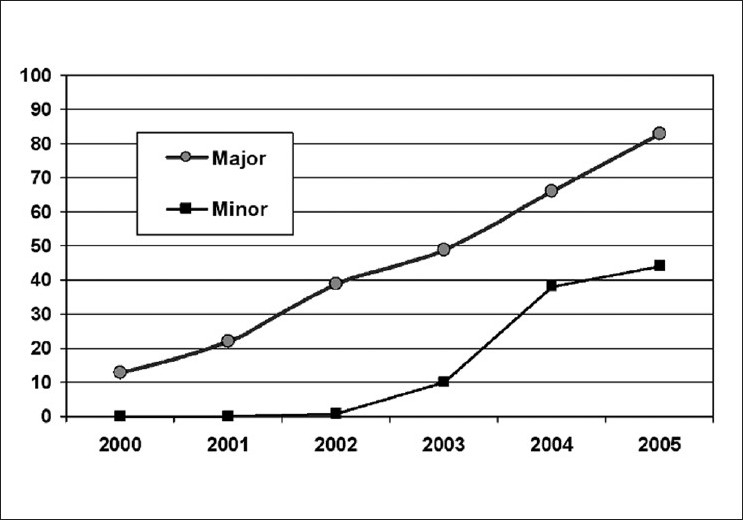
The increasing rate of phacoemulsification in Iran at major and minor cataract surgery units from 2000 to 2005. The technique was adopted at minor centers after 2002, but there seems to have been a similar growth rate

ICCE was performed in 0.4%, lensectomy was performed in 2.0%, phacoemulsification was performed in 47.3%, or ECCE was performed in 50.4%, of the cases, over the 6-year span. Table 2 documents the yearly percentages of cataract surgery. These figures indicate a statistically significant change in the rate of ECCE and phacoemulsification from 2000 to 2005 (*P*<0.0001). Figure 1 demonstrates the increasing rate of phacoemulsification at both major and minor cataract surgery centers.

**Table 2:** Percentage rates of each type of cataract surgery performed yearly

	ICCE*	Lensectomy	ECCEҰ	Phaco‡
2000	0.69	0.84	91.63	6.85
2001	0.21	0.93	90.59	8.26
2002	0.11	01.33	83.11	15.45
2003	0.27	01.31	72.82	25.60
2004	0.11	0.86	49.77	49.26
2005	0.65	1.44	41.04	56.86
*P* value	0.047	0.475	< 0.0001	< 0.0001

* Intracapsular cataract extraction

Ұ Extracapsulat cataract extraction

‡ Phacoemulsification

**Figure 2:**
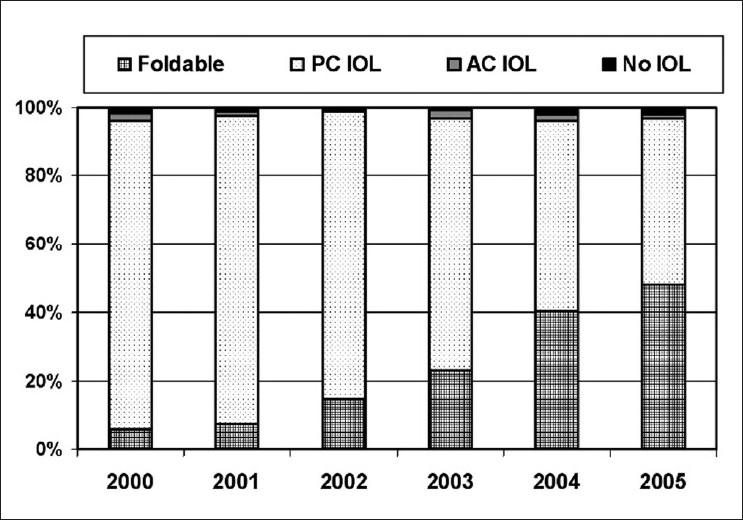
Change in the choice of intraocular lenses in Iran between 2000 and 2005

Figure 2 illustrates the change in the choice of IOLs. Over 6 years, the use of foldable IOLs increased statistically significantly from 10.9% in 2000 to 67.3% in 2005 [Figure 2] (*P*<0.0001). In 2005, IOLs were implanted in 97.9% cases of cataract surgeries performed throughout Iran.

## DISCUSSION

The first phacoemulsification procedure was performed in 1967, and gained popularity in developed countries during the 1980s. In Iran, however, phacoemulsification was first performed in 1993 at Farabi Eye Hospital,^13^ a teaching eye hospital affiliated with Tehran University of Medical Sciences, and simultaneously at Shoorideh Medical Center, a private center in Tehran.

The results of the present study indicate that phacoemulsification with IOL implantation has progressively become the most common type of cataract surgery performed in Iran. For example, the overall rate of phacoemulsification with IOL implantation was 6.85% in 2000 and increased to 57% in 2005. In 2000, few cornea fellows were officially trained to perform the procedure. Phacoemulsification was included in the residency program curriculum at Farabi Eye Hospital, Teheran in 2001. Despite the recent introduction of phacoemulsification in 2005, more than 80% of cataract surgical procedures in major centers performed with phacoemulsification and IOL implantation indicating the popularity of this procedure among surgeons. This increase is assumed to be associated with better outcomes.^14^ We suggest that the rate of phacoemulsification to perform cataract surgery will further increase especially at smaller centers since affordable phacoemulsification units and consumables are currently being produced and distributed in Iran. h. In addition, Iranian insurance policies have begun to cover the costs of surgery and the different types of IOLs.

However, certain barriers to adoption of phacoemulsification might exist and such barriers may not necessarily affect surgical outcomes. For example, hypermature or dense cataracts may be more prevalent in developing countries^8^,^15^ which may necessitate attempting alternate surgical procedures to avoid complications. For instance, manual small incision cataract surgery (SICS) is as safe and effective yet faster and less expensive than phacoemulsification.^16^ Phacoemulsification also requires surgeons who are willing to receive training and go through a transition process.

One limitation of our study is the lack of data on SICS. Another limitation was the inclusion of all surgical cases, irrespective of their visual acuity. This may have contributed to a slight overestimation of the CSR, although patients with blinding cataract receive higher priority in Iran. Another limitation was the lack of evaluation of long-term results of different techniques or implanted IOLs. However the goal of the current study was to report the general trends of cataract surgical techniques in Iran, not a comprehensive review of each technique. We recommend future studies that address the limitations of this study as they may provide additional data for health policy makers and for the Vision 2020 initiative.

Irrespective of the type of cataract surgery, the Vision 2020 initiative recommends IOLs to be implanted in every case, unless contraindicated. Leaving the patient dependent upon aphakic spectacles often results in noncompliance due to image magnification, physical discomfort, and limited visual field. Studies have shown that there remains a significant demand for spectacles for refractive errors and aphakia.^17^–^23^ Therefore, surgical methods such as secondary IOL implantation have been proposed for aphakes.^24^ According to our survey, IOLs were used in 97.9% of cataract surgeries performed in 2005.

Based on our results we recommend a study of the long-term outcomes of IOL implantation in Iran. With the advent of multifocal IOLs, the elderly undergoing cataract surgery can hope to be completely spectacle free. Although IOL implantation is one of the most successful surgical procedures in medicine and complication free in the majority of cases, opacification may occur during the postoperative period. As Trivedi *et al*.^25^ state, opacification may occur in front of, on, within, between and behind different types of IOLs. This indicates that further improvements in IOL design and material are required to achieve the goals of Vision 2020.

## CONCLUSION

Phacoemulsification with IOL implantation, although new in Iran, has become the most common technique for cataract surgery. Further studies are needed to evaluate progress toward the goals of the Vision 2020 initiative in terms of service quality, postoperative visual outcomes, and the quality of vision, specifically to monitor the rising trend in phacoemulsification with IOL implantation in Iran.
